# *Anchusellacretica* (Boraginaceae): a new genus and species record for the flora of Kosovo (Southeast Europe)

**DOI:** 10.3897/BDJ.13.e164900

**Published:** 2025-08-15

**Authors:** Elez Krasniqi, Naim Berisha

**Affiliations:** 1 Department of Biology, Faculty of Mathematics and Natural Sciences, University of Prishtina, Kosovo, Prishtina, Kosovo Department of Biology, Faculty of Mathematics and Natural Sciences, University of Prishtina, Kosovo Prishtina Kosovo

## Abstract

**Background:**

*Anchusellacretica* (Boraginaceae) is recorded for the first time in Kosovo and represents a new genus and species for the national flora. This range extension improves the knowledge about floristic and biogeographic patterns in the Western Balkans. Based on fieldwork in 2024–2025, four distinct populations were identified in dry, rocky, calcareous grasslands of the sub-Mediterranean region. The species shows a limited extent of occurrence (EOO = 80 km²) and area of occupancy (AOO = 5.54 km²) is limited. It is therefore assessed as Near Threatened (NT) at national level according to IUCN criteria. This finding emphasizes the importance of further botanical surveys and the conservation of underexplored lowland habitats in Kosovo.

**New information:**

new records, flora, Boraginaceae, distribution, range extension, Balkans

## Introduction

The Mediterranean basin is one of the richest regions in the world in terms of floristic diversity, harbouring numerous endemic and biogeographically important taxa (Cowling et al. 1996, Medail and Quezel 1997). This region is considered a hotspot of phytodiversity, hosting an extraordinary richness of over 20.000 vascular plant species, according to Schultz (1995). Within this region, the Boraginaceae family, particularly the tribe Boragineae Bercht. & J. Presl, exhibits considerable taxonomic complexity (Cohen 2013, Chacón et al. 2016), with several genera historically grouped under the broad term *Anchusa* L. *sensu lato* (Selvi and Bigazzi 1998, Hilger et al. 2004). From these, *Anchusella* Bigazzi, E. Nardi & Selvi was separated in the late 20th century on the basis of a combination of morphological, karyological, palynological and phylogenetic evidence (Bigazzi et al. 1997) to accommodate two annual species formerly belonging to *Anchusa*: *Anchusellacretica* (Mill.) Bigazzi, E. Nardi & Selvi and *Anchusellavariegata* (L.) Bigazzi, E. Nardi & Selvi.

*Anchusellacretica* is an annual species from the eastern Mediterranean, characterised by an annual growth habit, a curved purple to bluish corolla with five unequal lobes and nutlets with a distinctly reticulate surface (Bigazzi et al. 1997). It is typically found in dry, open, rocky habitats with light-requiring vegetation and has shown a preference for calcareous or occasionally siliceous substrates (Demiri 1983, Matevski 2010, Dimopoulos et al. 2013, Chytrý et al. 2024). Phytogeographically, the species is widespread in the central-eastern Mediterranean region, quite continuously distributed across Greece (Strid 2024, but not so in southern and central Italy, the Adriatic coast and parts of the western Balkans (Pils 2016, Barina et al. 2018, Anonymous 2025a). Occurrence data from GBIF - Global Biodiversity Information Facility (Anonymous 2025b) confirm its presence in Greece (444 records), Italy (215), Croatia (86), Albania (29) and slightly in Montenegro, Bosnia & Herzegovina and North Macedonia. The species is also documented in local floras such as those of Greece (Dimopoulos et al. 2013, Gutermann et al. 2014, Strid et al. 2017), Albania (Barina et al. 2018, Vangjeli 2018), North Macedonia (Matevski 2010) and the eastern Adriatic coast in Croatia (Bogdanović and Nikolić 2014).

Molecular phylogenetic analyses of the tribe Boragineae, based on nuclear ITS1 (internal transcribed spacer 1, a non-coding region of nuclear ribosomal DNA commonly used in phylogenetic studies) and chloroplast trnL sequences (a non-coding intron region in the chloroplast genome often used for plant phylogenetics), have supported the monophyly of *Anchusella* and its separation from *Anchusa* s.str., *Hormuzakia*, and *Cynoglottis*, among others (Hilger et al. 2004). In these analyses, *Anchusella* appears within a distinct clade that shows consistent molecular and morphological cohesion and limited intraspecific variation despite a relatively wide geographic distribution range (Hilger et al. 2004).

Although *A.cretica* is widely distributed in the central and eastern Mediterranean (Matevski 2010, Cohen 2013, Bogdanović and Nikolić 2014, Chacón et al. 2016, Strid et al. 2017, Barina et al. 2018, Vangjeli 2018), it has never been reported from the flora of Kosovo yet. During recent botanical field studies in south-western Kosovo, individuals morphologically matching *A.cretica* were discovered in dry, open, rocky grasslands on limestone substrates. The specimens were collected and identified on the basis of the most important morphological features and compared with the relevant taxonomic descriptions (Bigazzi et al. 1997, Selvi and Bigazzi 1998).

This study represents the first confirmed record of *Anchusellacretica* in Kosovo and thus marks a remarkable extension of the known distribution range of this species. It provides detailed data on the location, habitat conditions, and ecological preferences of the species and offers new insights into the flora of the region. In addition, the presence of *A.cretica* in Kosovo supports phytogeographical links between the western Balkans and the Italian Peninsula. By documenting this occurrence, the study contributes to a more comprehensive understanding of the floristic composition and biogeographical patterns of the Balkan Peninsula.

## Materials and methods

The occurrence of *Anchusellacretica* in Kosovo was initially documented by chance during botanical field surveys, conducted as part of habitat-based surveys focused on calcareous grasslands in the southern part of the country in spring 2024. Following this discovery, targeted field research was conducted to assess the local distribution, habitat characteristics, population size, and ecological preferences of the species.

The study area was visited several times during the 2024 as well as the 2025 growing seasons to ensure accurate documentation of phenological stages and site conditions. During these visits, data were collected on the species’ substrate type, vegetation structure, exposure, elevation, and associated plant communities. The latter were characterized by open, semi-dry calcareous grasslands belonging to the vegetation class: *Bromionerecti* Koch 1926 as well as *Festuco*-*Brometea* Br.-Bl. et Tx. ex Soó 1947 (probably within the alliance *Scorzonerionvillosae* Horvatić ex Kovačević 1959), with associated species such as *Euphorbiacyparissias* L., *Erodiumcicutarium* (L.) L'Hér., *Veronica* spp, *Capsellabursa-pastoris* (L.) Medik. and *Drabaverna* L., among others. Population estimates were made by direct counts of individuals within the marked plots and habitat conditions were assessed by qualitative observation and GPS-referenced mapping. To estimate population density, we used a quadrat method in which we randomly marked out 2×2 m plots in the occupied habitat. Within each quadrant, all visible individuals of *A.cretica* were counted. Density was calculated as the total number of individuals divided by the total sampled area (individuals/m²) according to standard floristic survey protocols (Stehman and Salzer 2000).

Representative specimens were collected, pressed, and mounted as herbarium specimens. They are now deposited in the herbarium of the Faculty of Mathematics and Natural Sciences of the University of Prishtina (UPH). Identification was based on diagnostic morphological characters described in the original taxonomic literature (Bigazzi et al. 1997, Selvi and Bigazzi 1998) and verified by comparison with published floristic works and herbarium specimens.

## Taxon treatments

### Anchusella cretica

(Mill.) Bigazzi, E. Nardi & Selvi

4E51815D-D4C2-546F-91D5-769A3086A2CF

urn:lsid:ipni.org:names:995790-1

#### Materials

- **Type status:** Other material. **Occurrence:** occurrenceRemarks: New national record; recordedBy: Krasniqi & Berisha; occurrenceID: 25B7C46E-906D-5A9F-87F9-5D40147BCB7F; **Taxon:** taxonID: urn:lsid:ipni.org:names:995790-1; originalNameUsageID: urn:lsid:ipni.org:names:995790-1; scientificName: Anchusellacretica (Mill.) Bigazzi & al. in Pl. Syst. Evol. 205: 257. 1997; acceptedNameUsage: Anchusellacretica (Mill.) Bigazzi & al.; parentNameUsage: Boraginaceae; originalNameUsage: Anchusacretica Mill., Gard. Dict., ed. 8: Anchusa no. 7. 1768; kingdom: Plantae; phylum: Spermatophytina; class: Magnoliopsida; order: Boraginales; family: Boraginaceae; genus: Anchusella; taxonRank: Species; scientificNameAuthorship: (Mill.) Bigazzi, E. Nardi & Selvi; nomenclaturalCode: urn:lsid:ipni.org:names:995790-1; taxonomicStatus: Accepted; **Location:** higherGeographyID: SE Europe; higherGeography: SE Europe; continent: Europe; country: Kosovo; countryCode: XK; municipality: Prizren; locality: Lugina e Drinit; verbatimElevation: 315 m; verbatimCoordinates: 42.227927°N, 20.639507°E; verbatimLatitude: 42.227927°N; verbatimLongitude: 20.639507°E; georeferenceProtocol: GPS; **Identification:** identifiedBy: Naim Berisha; dateIdentified: 3/31/2025; **Event:** eventDate: 3/31/2025; **Record Level:** language: en; collectionCode: Plants; basisOfRecord: PreservedSpecimen (Herbarium)
- **Type status:** Other material. **Occurrence:** occurrenceRemarks: New national record; recordedBy: Krasniqi & Berisha; occurrenceID: 114AC267-1902-50CD-AF32-241030B3F9FE; **Taxon:** taxonID: urn:lsid:ipni.org:names:995790-1; originalNameUsageID: urn:lsid:ipni.org:names:995790-1; scientificName: Anchusellacretica (Mill.) Bigazzi & al. in Pl. Syst. Evol. 205: 257. 1997; acceptedNameUsage: Anchusellacretica (Mill.) Bigazzi & al.; parentNameUsage: Boraginaceae; originalNameUsage: Anchusacretica Mill., Gard. Dict., ed. 8: Anchusa no. 7. 1768; kingdom: Plantae; phylum: Spermatophytina; class: Magnoliopsida; order: Boraginales; family: Boraginaceae; genus: Anchusella; taxonRank: Species; scientificNameAuthorship: (Mill.) Bigazzi, E. Nardi & Selvi; nomenclaturalCode: urn:lsid:ipni.org:names:995790-1; taxonomicStatus: Accepted; **Location:** higherGeographyID: SE Europe; higherGeography: SE Europe; continent: Europe; country: Kosovo; countryCode: XK; municipality: Prizren; locality: Lugina e Drinit; verbatimElevation: 315 m; verbatimCoordinates: 42.226860°N, 20.638344°E; verbatimLatitude: 42.226860°N; verbatimLongitude: 20.638344°E; georeferenceProtocol: GPS; **Identification:** identifiedBy: Naim Berisha; dateIdentified: 3/31/2025; **Event:** eventDate: 3/31/2025; **Record Level:** language: en; collectionCode: Plants; basisOfRecord: PreservedSpecimen (Herbarium)
- **Type status:** Other material. **Occurrence:** occurrenceRemarks: New national record; recordedBy: Krasniqi & Berisha; occurrenceID: 79AFFBEB-A6B0-55F4-BA52-D4A9D20B80DD; **Taxon:** taxonID: urn:lsid:ipni.org:names:995790-1; originalNameUsageID: urn:lsid:ipni.org:names:995790-1; scientificName: Anchusellacretica (Mill.) Bigazzi & al. in Pl. Syst. Evol. 205: 257. 1997; acceptedNameUsage: Anchusellacretica (Mill.) Bigazzi & al.; parentNameUsage: Boraginaceae; originalNameUsage: Anchusacretica Mill., Gard. Dict., ed. 8: Anchusa no. 7. 1768; kingdom: Plantae; phylum: Spermatophytina; class: Magnoliopsida; order: Boraginales; family: Boraginaceae; genus: Anchusella; taxonRank: Species; scientificNameAuthorship: (Mill.) Bigazzi, E. Nardi & Selvi; nomenclaturalCode: urn:lsid:ipni.org:names:995790-1; taxonomicStatus: Accepted; **Location:** higherGeographyID: SE Europe; higherGeography: SE Europe; continent: Europe; country: Kosovo; countryCode: XK; municipality: Prizren; locality: Lugina e Drinit; verbatimElevation: 315 m; verbatimCoordinates: 42.221352°N, 20.637665°E; verbatimLatitude: 42.221352°N; verbatimLongitude: 20.637665°E; georeferenceProtocol: GPS; **Identification:** identifiedBy: Naim Berisha; dateIdentified: 3/31/2025; **Event:** eventDate: 3/31/2025; **Record Level:** language: en; collectionCode: Plants; basisOfRecord: PreservedSpecimen (Herbarium)
- **Type status:** Other material. **Occurrence:** occurrenceRemarks: New national record; recordedBy: Krasniqi & Berisha; occurrenceID: 8ECF137E-2D5C-5FD7-A68C-F40770CD9862; **Taxon:** taxonID: urn:lsid:ipni.org:names:995790-1; originalNameUsageID: urn:lsid:ipni.org:names:995790-1; scientificName: Anchusellacretica (Mill.) Bigazzi & al. in Pl. Syst. Evol. 205: 257. 1997; acceptedNameUsage: Anchusellacretica (Mill.) Bigazzi & al.; parentNameUsage: Boraginaceae; originalNameUsage: Anchusacretica Mill., Gard. Dict., ed. 8: Anchusa no. 7. 1768; kingdom: Plantae; phylum: Spermatophytina; class: Magnoliopsida; order: Boraginales; family: Boraginaceae; genus: Anchusella; taxonRank: Species; scientificNameAuthorship: (Mill.) Bigazzi, E. Nardi & Selvi; nomenclaturalCode: urn:lsid:ipni.org:names:995790-1; taxonomicStatus: Accepted; **Location:** higherGeographyID: SE Europe; higherGeography: SE Europe; continent: Europe; country: Kosovo; countryCode: XK; municipality: Prizren; locality: Lugina e Drinit; verbatimElevation: 315 m; verbatimCoordinates: 42.217452°N, 20.630672°E; verbatimLatitude: 42.217452°N; verbatimLongitude: 20.630672°E; georeferenceProtocol: GPS; **Identification:** identifiedBy: Naim Berisha; dateIdentified: 3/31/2025; **Event:** eventDate: 3/31/2025; **Record Level:** language: en; collectionCode: Plants; basisOfRecord: PreservedSpecimen (Herbarium)
- **Type status:** Other material. **Occurrence:** occurrenceRemarks: New national record; recordedBy: Krasniqi & Berisha; occurrenceID: D9659D6E-321F-5A24-A00C-9674091CE635; **Taxon:** taxonID: urn:lsid:ipni.org:names:995790-1; originalNameUsageID: urn:lsid:ipni.org:names:995790-1; scientificName: Anchusellacretica (Mill.) Bigazzi & al. in Pl. Syst. Evol. 205: 257. 1997; acceptedNameUsage: Anchusellacretica (Mill.) Bigazzi & al.; parentNameUsage: Boraginaceae; originalNameUsage: Anchusacretica Mill., Gard. Dict., ed. 8: Anchusa no. 7. 1768; kingdom: Plantae; phylum: Spermatophytina; class: Magnoliopsida; order: Boraginales; family: Boraginaceae; genus: Anchusella; taxonRank: Species; scientificNameAuthorship: (Mill.) Bigazzi, E. Nardi & Selvi; nomenclaturalCode: urn:lsid:ipni.org:names:995790-1; taxonomicStatus: Accepted; **Location:** higherGeographyID: SE Europe; higherGeography: SE Europe; continent: Europe; country: Kosovo; countryCode: XK; municipality: Prizren; locality: Planeje; verbatimElevation: 708 m; verbatimCoordinates: 42.215771°N, 20.576035°E; verbatimLatitude: 42.215771°N; verbatimLongitude: 20.576035°E; georeferenceProtocol: GPS; **Identification:** identifiedBy: Naim Berisha; dateIdentified: 3/31/2025; **Event:** eventDate: 3/31/2025; **Record Level:** language: en; collectionCode: Plants; basisOfRecord: PreservedSpecimen (Herbarium)
- **Type status:** Other material. **Occurrence:** occurrenceRemarks: New national record; recordedBy: Krasniqi & Berisha; occurrenceID: AFED05C5-A299-5B4C-B00E-FED92B66E67E; **Taxon:** taxonID: urn:lsid:ipni.org:names:995790-1; originalNameUsageID: urn:lsid:ipni.org:names:995790-1; scientificName: Anchusellacretica (Mill.) Bigazzi & al. in Pl. Syst. Evol. 205: 257. 1997; acceptedNameUsage: Anchusellacretica (Mill.) Bigazzi & al.; parentNameUsage: Boraginaceae; originalNameUsage: Anchusacretica Mill., Gard. Dict., ed. 8: Anchusa no. 7. 1768; kingdom: Plantae; phylum: Spermatophytina; class: Magnoliopsida; order: Boraginales; family: Boraginaceae; genus: Anchusella; taxonRank: Species; scientificNameAuthorship: (Mill.) Bigazzi, E. Nardi & Selvi; nomenclaturalCode: urn:lsid:ipni.org:names:995790-1; taxonomicStatus: Accepted; **Location:** higherGeographyID: SE Europe; higherGeography: SE Europe; continent: Europe; country: Kosovo; countryCode: XK; municipality: Prizren; locality: Planeje; verbatimElevation: 708 m; verbatimCoordinates: 42.215079°N, 20.575552°E; verbatimLatitude: 42.215079°N; verbatimLongitude: 20.575552°E; georeferenceProtocol: GPS; **Identification:** identifiedBy: Naim Berisha; dateIdentified: 3/31/2025; **Event:** eventDate: 3/31/2025; **Record Level:** language: en; collectionCode: Plants; basisOfRecord: PreservedSpecimen (Herbarium)
- **Type status:** Other material. **Occurrence:** occurrenceRemarks: New national record; recordedBy: Krasniqi & Berisha; occurrenceID: 9FC4CC2A-EC2E-5A55-A93B-5CF440249645; **Taxon:** taxonID: urn:lsid:ipni.org:names:995790-1; originalNameUsageID: urn:lsid:ipni.org:names:995790-1; scientificName: Anchusellacretica (Mill.) Bigazzi & al. in Pl. Syst. Evol. 205: 257. 1997; acceptedNameUsage: Anchusellacretica (Mill.) Bigazzi & al.; parentNameUsage: Boraginaceae; originalNameUsage: Anchusacretica Mill., Gard. Dict., ed. 8: Anchusa no. 7. 1768; kingdom: Plantae; phylum: Spermatophytina; class: Magnoliopsida; order: Boraginales; family: Boraginaceae; genus: Anchusella; taxonRank: Species; scientificNameAuthorship: (Mill.) Bigazzi, E. Nardi & Selvi; nomenclaturalCode: urn:lsid:ipni.org:names:995790-1; taxonomicStatus: Accepted; **Location:** higherGeographyID: SE Europe; higherGeography: SE Europe; continent: Europe; country: Kosovo; countryCode: XK; municipality: Prizren; locality: Planeje; verbatimElevation: 708 m; verbatimCoordinates: 42.215276°N, 20.577337°E; verbatimLatitude: 42.215276°N; verbatimLongitude: 20.577337°E; georeferenceProtocol: GPS; **Identification:** identifiedBy: Naim Berisha; dateIdentified: 3/31/2025; **Event:** eventDate: 3/31/2025; **Record Level:** language: en; collectionCode: Plants; basisOfRecord: PreservedSpecimen (Herbarium)
- **Type status:** Other material. **Occurrence:** occurrenceRemarks: New national record; recordedBy: Krasniqi & Berisha; occurrenceID: 3209874E-AD13-59D7-8FED-51F82CCBB7C6; **Taxon:** taxonID: urn:lsid:ipni.org:names:995790-1; originalNameUsageID: urn:lsid:ipni.org:names:995790-1; scientificName: Anchusellacretica (Mill.) Bigazzi & al. in Pl. Syst. Evol. 205: 257. 1997; acceptedNameUsage: Anchusellacretica (Mill.) Bigazzi & al.; parentNameUsage: Boraginaceae; originalNameUsage: Anchusacretica Mill., Gard. Dict., ed. 8: Anchusa no. 7. 1768; kingdom: Plantae; phylum: Spermatophytina; class: Magnoliopsida; order: Boraginales; family: Boraginaceae; genus: Anchusella; taxonRank: Species; scientificNameAuthorship: (Mill.) Bigazzi, E. Nardi & Selvi; nomenclaturalCode: urn:lsid:ipni.org:names:995790-1; taxonomicStatus: Accepted; **Location:** higherGeographyID: SE Europe; higherGeography: SE Europe; continent: Europe; country: Kosovo; countryCode: XK; municipality: Prizren; locality: Planeje; verbatimElevation: 708 m; verbatimCoordinates: 42.209428°N, 20.579546°E; verbatimLatitude: 42.209428°N; verbatimLongitude: 20.579546°E; georeferenceProtocol: GPS; **Identification:** identifiedBy: Naim Berisha; dateIdentified: 3/31/2025; **Event:** eventDate: 3/31/2025; **Record Level:** language: en; collectionCode: Plants; basisOfRecord: PreservedSpecimen (Herbarium)
- **Type status:** Other material. **Occurrence:** occurrenceRemarks: New national record; recordedBy: Krasniqi & Berisha; occurrenceID: DAD2B87F-BC36-5516-BB19-BE4D13886D8B; **Taxon:** taxonID: urn:lsid:ipni.org:names:995790-1; originalNameUsageID: urn:lsid:ipni.org:names:995790-1; scientificName: Anchusellacretica (Mill.) Bigazzi & al. in Pl. Syst. Evol. 205: 257. 1997; acceptedNameUsage: Anchusellacretica (Mill.) Bigazzi & al.; parentNameUsage: Boraginaceae; originalNameUsage: Anchusacretica Mill., Gard. Dict., ed. 8: Anchusa no. 7. 1768; kingdom: Plantae; phylum: Spermatophytina; class: Magnoliopsida; order: Boraginales; family: Boraginaceae; genus: Anchusella; taxonRank: Species; scientificNameAuthorship: (Mill.) Bigazzi, E. Nardi & Selvi; nomenclaturalCode: urn:lsid:ipni.org:names:995790-1; taxonomicStatus: Accepted; **Location:** higherGeographyID: SE Europe; higherGeography: SE Europe; continent: Europe; country: Kosovo; countryCode: XK; municipality: Prizren; locality: Mazrek; verbatimElevation: 360 m; verbatimCoordinates: 42.230282°N, 20.616600°E; verbatimLatitude: 42.230282°N; verbatimLongitude: 20.616600°E; georeferenceProtocol: GPS; **Identification:** identifiedBy: Naim Berisha; dateIdentified: 3/31/2025; **Event:** eventDate: 3/31/2025; **Record Level:** language: en; collectionCode: Plants; basisOfRecord: PreservedSpecimen (Herbarium)
- **Type status:** Other material. **Occurrence:** occurrenceRemarks: New national record; recordedBy: Krasniqi & Berisha; occurrenceID: EF62FA45-37E4-5071-BC1B-5F3A2B005F05; **Taxon:** taxonID: urn:lsid:ipni.org:names:995790-1; originalNameUsageID: urn:lsid:ipni.org:names:995790-1; scientificName: Anchusellacretica (Mill.) Bigazzi & al. in Pl. Syst. Evol. 205: 257. 1997; acceptedNameUsage: Anchusellacretica (Mill.) Bigazzi & al.; parentNameUsage: Boraginaceae; originalNameUsage: Anchusacretica Mill., Gard. Dict., ed. 8: Anchusa no. 7. 1768; kingdom: Plantae; phylum: Spermatophytina; class: Magnoliopsida; order: Boraginales; family: Boraginaceae; genus: Anchusella; taxonRank: Species; scientificNameAuthorship: (Mill.) Bigazzi, E. Nardi & Selvi; nomenclaturalCode: urn:lsid:ipni.org:names:995790-1; taxonomicStatus: Accepted; **Location:** higherGeographyID: SE Europe; higherGeography: SE Europe; continent: Europe; country: Kosovo; countryCode: XK; municipality: Prizren; locality: Mazrek; verbatimElevation: 360 m; verbatimCoordinates: 42.233447°N, 20.617197°E; verbatimLatitude: 42.233447°N; verbatimLongitude: 20.617197°E; georeferenceProtocol: GPS; **Identification:** identifiedBy: Naim Berisha; dateIdentified: 3/31/2025; **Event:** eventDate: 3/31/2025; **Record Level:** language: en; collectionCode: Plants; basisOfRecord: PreservedSpecimen (Herbarium)
- **Type status:** Other material. **Occurrence:** occurrenceRemarks: New national record; recordedBy: Krasniqi & Berisha; occurrenceID: 7203DA2A-5473-5EB1-9ABF-2B4389870630; **Taxon:** taxonID: urn:lsid:ipni.org:names:995790-1; originalNameUsageID: urn:lsid:ipni.org:names:995790-1; scientificName: Anchusellacretica (Mill.) Bigazzi & al. in Pl. Syst. Evol. 205: 257. 1997; acceptedNameUsage: Anchusellacretica (Mill.) Bigazzi & al.; parentNameUsage: Boraginaceae; originalNameUsage: Anchusacretica Mill., Gard. Dict., ed. 8: Anchusa no. 7. 1768; kingdom: Plantae; phylum: Spermatophytina; class: Magnoliopsida; order: Boraginales; family: Boraginaceae; genus: Anchusella; taxonRank: Species; scientificNameAuthorship: (Mill.) Bigazzi, E. Nardi & Selvi; nomenclaturalCode: urn:lsid:ipni.org:names:995790-1; taxonomicStatus: Accepted; **Location:** higherGeographyID: SE Europe; higherGeography: SE Europe; continent: Europe; country: Kosovo; countryCode: XK; municipality: Prizren; locality: Ura e Fshajte; verbatimElevation: 310 m; verbatimCoordinates: 42.354280°N, 20.542625°E; verbatimLatitude: 42.354280°N; verbatimLongitude: 20.542625°E; georeferenceProtocol: GPS; **Identification:** identifiedBy: Naim Berisha; dateIdentified: 3/31/2025; **Event:** eventDate: 3/31/2025; **Record Level:** language: en; collectionCode: Plants; basisOfRecord: PreservedSpecimen (Herbarium)
- **Type status:** Other material. **Occurrence:** occurrenceRemarks: New national record; recordedBy: Krasniqi & Berisha; occurrenceID: 6B775A8B-8204-5DF0-9789-AA8B5E2FAE04; **Taxon:** taxonID: urn:lsid:ipni.org:names:995790-1; originalNameUsageID: urn:lsid:ipni.org:names:995790-1; scientificName: Anchusellacretica (Mill.) Bigazzi & al. in Pl. Syst. Evol. 205: 257. 1997; acceptedNameUsage: Anchusellacretica (Mill.) Bigazzi & al.; parentNameUsage: Boraginaceae; originalNameUsage: Anchusacretica Mill., Gard. Dict., ed. 8: Anchusa no. 7. 1768; kingdom: Plantae; phylum: Spermatophytina; class: Magnoliopsida; order: Boraginales; family: Boraginaceae; genus: Anchusella; taxonRank: Species; scientificNameAuthorship: (Mill.) Bigazzi, E. Nardi & Selvi; nomenclaturalCode: urn:lsid:ipni.org:names:995790-1; taxonomicStatus: Accepted; **Location:** higherGeographyID: SE Europe; higherGeography: SE Europe; continent: Europe; country: Kosovo; countryCode: XK; municipality: Prizren; locality: Ura e Fshajte; verbatimElevation: 310 m; verbatimCoordinates: 42.354057°N, 20.540495°E; verbatimLatitude: 42.354057°N; verbatimLongitude: 20.540495°E; georeferenceProtocol: GPS; **Identification:** identifiedBy: Naim Berisha; dateIdentified: 3/31/2025; **Event:** eventDate: 3/31/2025; **Record Level:** language: en; collectionCode: Plants; basisOfRecord: PreservedSpecimen (Herbarium)
- **Type status:** Other material. **Occurrence:** occurrenceRemarks: New national record; recordedBy: Krasniqi & Berisha; occurrenceID: B1D118C9-7E4B-5F39-AB95-CD0B66265211; **Taxon:** taxonID: urn:lsid:ipni.org:names:995790-1; originalNameUsageID: urn:lsid:ipni.org:names:995790-1; scientificName: Anchusellacretica (Mill.) Bigazzi & al. in Pl. Syst. Evol. 205: 257. 1997; acceptedNameUsage: Anchusellacretica (Mill.) Bigazzi & al.; parentNameUsage: Boraginaceae; originalNameUsage: Anchusacretica Mill., Gard. Dict., ed. 8: Anchusa no. 7. 1768; kingdom: Plantae; phylum: Spermatophytina; class: Magnoliopsida; order: Boraginales; family: Boraginaceae; genus: Anchusella; taxonRank: Species; scientificNameAuthorship: (Mill.) Bigazzi, E. Nardi & Selvi; nomenclaturalCode: urn:lsid:ipni.org:names:995790-1; taxonomicStatus: Accepted; **Location:** higherGeographyID: SE Europe; higherGeography: SE Europe; continent: Europe; country: Kosovo; countryCode: XK; municipality: Prizren; locality: Ura e Fshajte; verbatimElevation: 310 m; verbatimCoordinates: 42.354392°N, 20.539100°E; verbatimLatitude: 42.354392°N; verbatimLongitude: 20.539100°E; georeferenceProtocol: GPS; **Identification:** identifiedBy: Naim Berisha; dateIdentified: 3/31/2025; **Event:** eventDate: 3/31/2025; **Record Level:** language: en; collectionCode: Plants; basisOfRecord: PreservedSpecimen (Herbarium)
- **Type status:** Other material. **Occurrence:** occurrenceRemarks: New national record; recordedBy: Krasniqi & Berisha; occurrenceID: C9BB3272-D045-58EA-ACF4-911AD14D7550; **Taxon:** taxonID: urn:lsid:ipni.org:names:995790-1; originalNameUsageID: urn:lsid:ipni.org:names:995790-1; scientificName: Anchusellacretica (Mill.) Bigazzi & al. in Pl. Syst. Evol. 205: 257. 1997; acceptedNameUsage: Anchusellacretica (Mill.) Bigazzi & al.; parentNameUsage: Boraginaceae; originalNameUsage: Anchusacretica Mill., Gard. Dict., ed. 8: Anchusa no. 7. 1768; kingdom: Plantae; phylum: Spermatophytina; class: Magnoliopsida; order: Boraginales; family: Boraginaceae; genus: Anchusella; taxonRank: Species; scientificNameAuthorship: (Mill.) Bigazzi, E. Nardi & Selvi; nomenclaturalCode: urn:lsid:ipni.org:names:995790-1; taxonomicStatus: Accepted; **Location:** higherGeographyID: SE Europe; higherGeography: SE Europe; continent: Europe; country: Kosovo; countryCode: XK; municipality: Prizren; locality: Ura e Fshajte; verbatimCoordinates: 42.355563°N, 20.545754°E; verbatimLatitude: 42.355563°N; verbatimLongitude: 20.545754°E; georeferenceProtocol: GPS; **Identification:** identifiedBy: Naim Berisha; dateIdentified: 3/31/2025; **Event:** eventDate: 3/31/2025; **Record Level:** language: en; collectionCode: Plants; basisOfRecord: PreservedSpecimen (Herbarium)

#### Distribution

**Figure 1.**
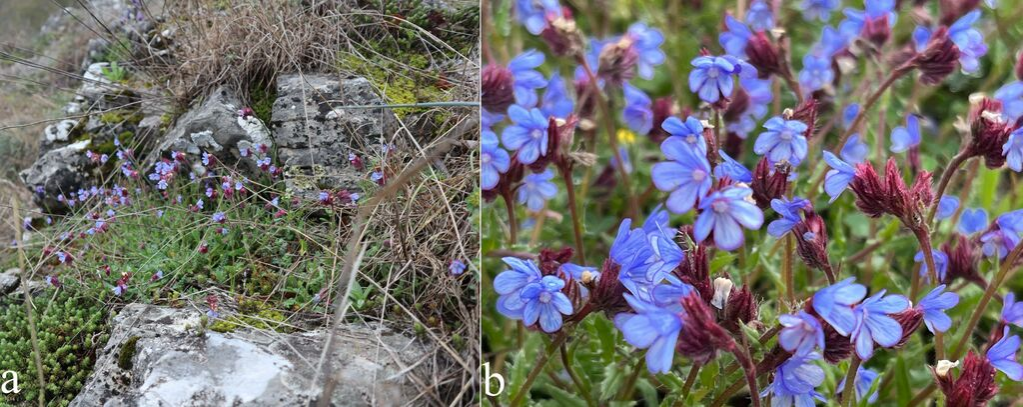
*Anchusellacretica* (Boraginaceae) in Kosovo. **a** Typical habitat on dry, rocky, calcareous slopes near the river Drini i Bardhë; **b** Close-up of the flowering individuals with the characteristic blue corolla and reddish calyx. (Photo: Berisha, N. - 31 March 2025).

**Figure 2.**
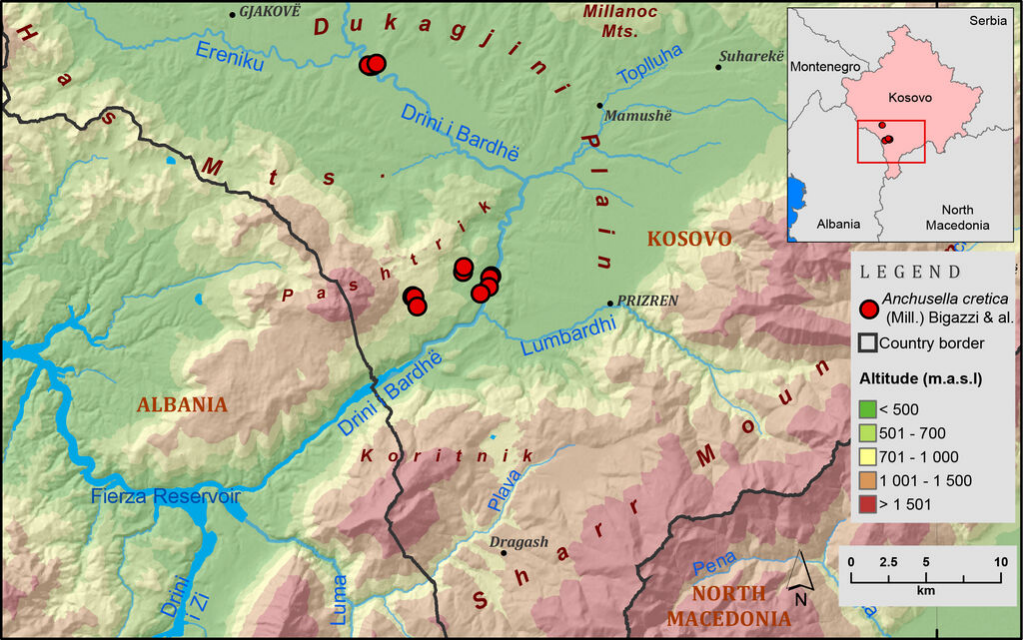
Confirmed distribution of *Anchusellacretica* in Kosovo based on field surveys.

In Kosovo, *Anchusellacretica* (Fig. 1) has been found in four disjunct populations of different sizes (Fig. 2). The first population consists of three subpopulations distributed along the western bank of the river Drini i Bardhë (Fig. 1), about 9.5 to 11.5 km north-east of the Albanian border. These sites are located on calcareous rocky slopes and dry, south-east facing clearings at altitudes between 306 and 328 meters above sea level.

The second population was found near the village of Planejë in the Hasi region, where *A.cretica* grows in dry, calcareous meadows that are apparently abandoned and no longer used for grazing or haymaking, at altitudes between 672 and 745 meters above sea level.

The third population occurs near the village of Mazrek (also in the Hasi region), where the species grows along roadsides in similar dry, calcareous meadow habitats. This site comprises two small subpopulations, each with only a few individuals (Table 1) - at altitudes between 470 and 490 meters above sea level.

The fourth and most remarkable population was discovered in the gorge of Ura e Fshajtë, on the upper bank of the river Drini i Bardhë. This population is not only the largest of those recorded, but also the most ecologically interesting. It inhabits steep, rocky, calcareous slopes and open clearings at altitudes between 340 and 360 meters above sea level. It is exposed to considerable human disturbance due to the nearby recreational infrastructure, including hiking trails and a hotel playground.

In terms of habitat classification (Anonymous 2023, Chytrý et al. 2024) according to the broad EUNIS system used by the European Union, the habitats where *Anchusellacretica* occurs in Kosovo fall predominantly under EUNIS habitat type E1.26 – Dry calcareous grasslands and steppes and partially overlap with EUNIS habitat H3.4 – South Balkan and Aegean calcareous screes on steep rocky slopes. These habitats are characterised by shallow, skeletal soils over limestone or dolomite, typically hosting a diverse assemblage of heliophilous and thermophilous species. Based on the FloraVeg.EU alliances (Chytrý et al. 2024), the vegetation broadly corresponds to *Bromionerecti* Koch 1926 and related dry grassland communities, often with *Festuco*-*Brometea* Br.-Bl. et Tx. ex Soó 1947 elements in transition. The occurrence of *A.cretica* in these habitats emphasises its preference for open, sun-exposed, nutrient-poor conditions and illustrates the ecological specificity of the species in the karstic lowlands and foothills of the central western Balkans.

#### Conservation

Based on currently available data, *Anchusellacretica* is known from four disjunct localities in southern Kosovo, with an estimated Area of Occupancy (AOO) of 5.54 km² and an Extent of Occurrence (EOO) of 80 km². These values are below the threshold values for the category "threatened" according to IUCN criterion B (Anonymous 2024). However, the development of the overall population, potential undiscovered sites and the exact impact of habitat conditions remain uncertain.

Although habitat disturbance has been observed in at least one major site (Ura e Fshajtë canyon), such open conditions may actually favour the persistence of this annual species by preventing encroachment from woody plants. This factor suggests that moderate disturbance does not pose an immediate threat and may even favour the persistence of the population.

Given these uncertainties, *A.cretica* is best categorised as Near Threatened (NT) at the national level in Kosovo, pending further data. This classification reflects the limited and fragmented distribution while recognising that the species may be more widespread than currently documented.

Given the recent discovery of the species in Kosovo and its ecological distinctiveness, *A.cretica* should be closely monitored together with targeted field surveys and habitat evaluations to clarify its conservation status and determine possible conservation measures.

## Discussion

**Figure 3.**
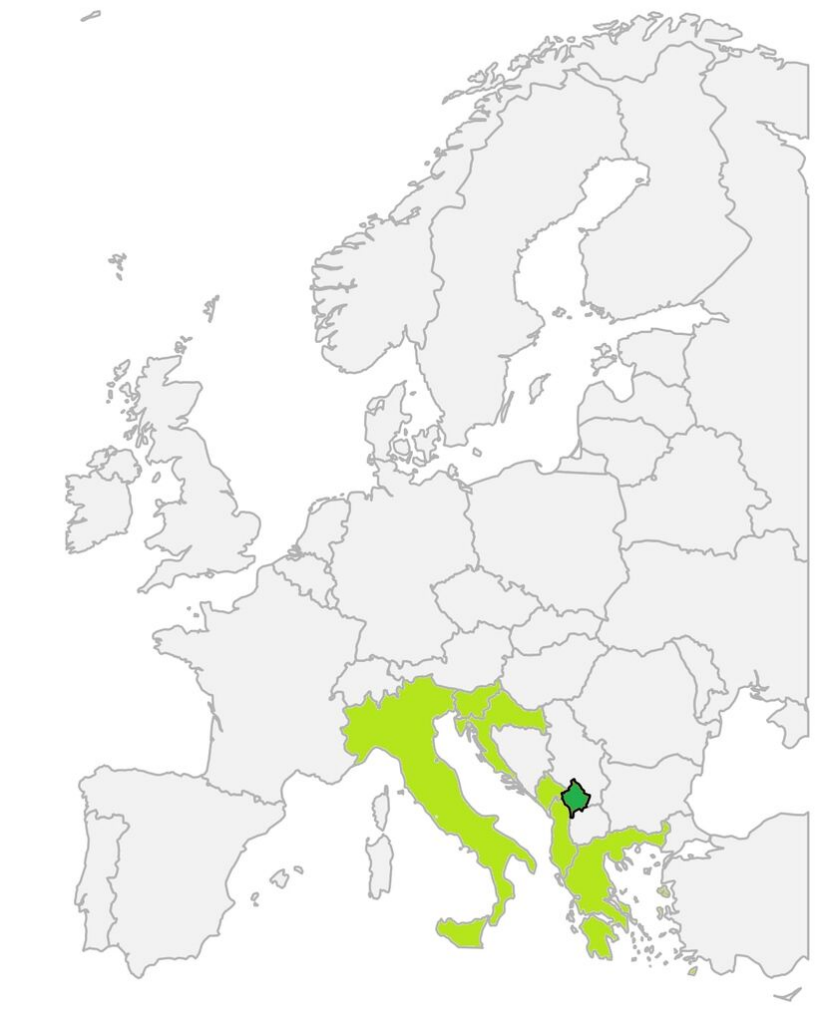
Known European distribution of *Anchusellacretica* with a new record from Kosovo indicated.

The present study reports on the first confirmed record of *Anchusellacretica* in the flora of Kosovo and represents a noteworthy extension of its known distribution range in the central-eastern Mediterranean. Prior to this report, the species was considered to be native to Greece, southern Italy, Albania and parts of the Adriatic coast including Montenegro, Croatia and Slovenia (Fig. 3) (Bigazzi et al. 1997, Bogdanović and Nikolić 2014, Pils 2016, Barina et al. 2018, Anonymous 2025a).

The discovery of four disjunct and ecologically distinct populations of *Anchusellacretica* in Kosovo not only fills a geographical gap, but also contributes to a better understanding of the biogeographical relationships in South-Eastern Europe (Berisha et al. 2025). These are likely to be previously overlooked populations, as the species occurs in floristically rich but historically understudied habitats due to limited botanical effort and funding. The regions where *A.cretica* has been found, although relatively easily accessible, have never been subjected to a detailed, systematic floristic surveys. It can therefore be assumed that the species has been present in this area for a long time, but has not yet been discovered.

The possibility of intentional or unintentional introduction by humans is considered highly unlikely. All recorded populations occur in natural or semi-natural habitats, far from urban centers, agricultural areas or disturbed sites that would typically be associated with anthropogenic dispersal. Furthermore, the species shows no signs of invasive behaviour or artificial aggregation that might indicate recent planting or escape.

Although a natural range expansion into Kosovo from nearby populations in Albania or Montenegro cannot be completely ruled out, especially under changing climate or land-use conditions, there are currently no data to support or refute this hypothesis. Given the fragmented but ecologically coherent distribution and the lack of direct dispersal vectors, the most plausible explanation is that *A.cretica* represents a native, previously undocumented component of the flora of Kosovo. Future studies, including long-term monitoring, may provide further insights into the historical biogeography and dispersal dynamics.

Ecologically, the observed habitats of *A.cretica* in Kosovo are very characteristic of the species’ known preferences for open, xeric, and calcareous environments (Chytrý et al. 2024).

The long overlooked occurrence of *A.cretica* in Kosovo can be attributed to its morphological similarity with certain *Anchusa* species, especially *Anchusaofficinalis*, which has a similar general habitus. This similarity has probably contributed to the species being misidentified in previous floristic surveys, especially in this region where a detailed taxonomic study is still lacking.

From a biogeographical point of view, all populations of *A.cretica* in Kosovo are located in the sub-Mediterranean region as recently defined by Berisha et al. (2025). This region represents a transition zone between the Mediterranean and the Continental region and is characterized by thermophilic vegetation and a mosaic of dry calcareous grasslands, rocky slopes and sparse forest edges. The occurrence of *A.cretica* in this zone is consistent with its general distribution pattern in south-eastern Europe and indicates that the ecological amplitude of the species is closely linked to these sub-Mediterranean habitat conditions, especially in karst landscapes at low to medium altitudes. The occurrence of the species in these habitats also underlines the floristic continuity between the limestone ecosystems of the western Balkans and those of the coastal and island regions of Albania and Greece.

From a conservation perspective, *Anchusellacretica* is only known from four disjunct populations in Kosovo, with a calculated Area of Occupancy (AOO) of 5.54 km² and an Extent of Occurrence (EOO) of 80 km². Although these values are below the thresholds typically associated with the "threatened" categories under IUCN Criterion B (Anonymous 2024), the current data are not sufficient to confirm ongoing decline or severe fragmentation. In addition, while habitat disturbance has been observed in at least one locality (e.g. from recreational activities in the Ura e Fshajtë canyon), these impacts may not pose an immediate threat to this annual species and may even favour its persistence by limiting woody encroachment. Based on these considerations, the species is best assessed as Near Threatened (NT) at the national level. This status emphasises the need for continuous monitoring, targeted field surveys and proactive habitat management to ensure the long-term viability of its populations.

### Conclusions

This study presents the first documented occurrence of *Anchusellacretica* in Kosovo, adding both a new species and a new genus to the national flora. Although the recorded populations are large in absolute numbers, they are spatially restricted to a few habitat patches on dry, rocky, calcareous slopes in the sub-Mediterranean biogeographic zone of south-western Kosovo. The limited extent of occurrence, highly fragmented distribution and habitat specificity indicate a relatively high vulnerability to environmental changes and human disturbances. These results support the categorisation of *A.cretica* as Near Threatened (NT) at the national level according to the IUCN Red List criteria. Apart from its taxonomic and conservation significance, the discovery contributes to a more nuanced understanding of the floristic and biogeographic links between the Adriatic coast and the Balkan interior. The long overlooked occurrence of the species, probably due to its similarity with *Anchusaofficinalis*, emphasizes the need for a more detailed taxonomic study of the overlooked lowland and sub-Mediterranean habitats in Kosovo. Future efforts should focus on habitat protection, further field surveys and the integration of this species into national conservation strategies.

Finally, the discovery of *A.cretica* in Kosovo highlights the need for continued botanical surveys in underexplored regions of the Balkans, where floristic richness and endemism are high, but documentation remains incomplete due to limited funding. This finding also contributes to a more precise understanding of the species’ European distribution and opens up future research opportunities on its ecological plasticity, pollination biology, and potential conservation applications in dry grassland management.

## Supplementary Material

XML Treatment for Anchusella cretica

## Figures and Tables

**Table 1.** Data on recorded *Anchusellacretica* populations in Kosovo.

**No**	**Locality**	**Elevation (m a.s.l.)**	**Habitat type**	**Population structure**	**GPS Coordinates**	**Density (ind. / m^2^)**	**Notes**
**1**	Banks of Drini i Bardhë	~ 315	Calcareous rocky slopes, SE exposure	3 sub-populations	LAT: 42.219633°N LON: 20.632487°E	20	Grazing minimal, natural vegetation intact
**2**	Planejë (Has)	~ 708	Dry calcareous meadows (abandoned)	Single	LAT: 42.215245°N LON: 20.575716°E	18	No current land use
**3**	Mazrek (Has)	~ 360	Roadside dry calcareous meadows	2 very small sub-popul.	LAT: 42.237886°N LON: 20.609693°E	11	Road maintenance may be a threat
**4**	Ura e Fshajtë canyon	~ 310	Rocky clearings, canyon slopes	Single (large)	LAT: 42.354367°N LON: 20.542787°E	18	Recreational disturbance near hotel
